# Cognitive Biases in Early Trauma Transfusions: Lessons From Three Rare Cases of Acute Hemolytic Reaction

**DOI:** 10.1002/ccr3.72944

**Published:** 2026-06-17

**Authors:** Mohammad Javad Entezari Meybodi, Samin Jodari Dallali, Hosein Kouchaki, Golnar Sabetian

**Affiliations:** ^1^ Student Research Committee, School of Medicine Shiraz University of Medical Sciences Shiraz Iran; ^2^ School of Pharmacy Inonu University Malatya Turkey; ^3^ Health Policy Research Center, Institute of Health Shiraz University of Medical Sciences Shiraz Iran; ^4^ Trauma Research Center Shiraz University of Medical Sciences Shiraz Iran

**Keywords:** blood transfusion, cognitive bias, critical care, diagnostic errors, hemolysis, hemolytic transfusion reaction, multiple trauma

## Abstract

Massive transfusion protocols are essential in trauma resuscitation but may inadvertently increase the risk of acute hemolytic transfusion reactions (AHTRs), which are typically caused by ABO incompatibility. Recognition is difficult because signs of hemolysis can mimic trauma‐related hemorrhage or coagulopathy. Cognitive biases in emergency decision‐making may further delay diagnosis. Although ABO‐incompatible AHTR remains exceptionally rare in trauma, it is easily overlooked in the context of polytrauma. We describe three cases of ABO‐incompatible AHTR admitted to a tertiary trauma center. First, a 52‐year‐old man with multiple injuries received three mis‐issued AB+ units instead of group O blood. He developed hemoglobinuria, coagulopathy, and fatal multiorgan failure. The second case involved an 81‐year‐old man with multiple comorbidities who underwent hip fracture fixation; due to a mislabeled admission sample, he received A+ instead of B+ blood, resulting in persistent anemia, renal failure, and death. The third case was a 30‐year‐old woman with severe head and thoracic trauma who received incompatible blood under an emergency release protocol. She developed hemolysis, neurologic decline, and sepsis, and ultimately died of multiorgan failure. In all three patients, early post‐transfusion anemia and hemodynamic instability were attributed to trauma, delaying recognition of hemolysis. AHTR may therefore be overlooked in trauma settings, where its manifestations overlap with injury‐related complications and cognitive biases such as anchoring and premature closure may impair diagnostic reasoning. Strict transfusion safety protocols, heightened vigilance when hemoglobin levels fail to rise, and awareness of cognitive errors may improve early recognition and patient outcomes.

## Key Clinical Message

In trauma resuscitation, acute hemolytic transfusion reactions (AHTRs) from ABO incompatibility, though rare, may be masked by hemorrhage and coagulopathy. We describe three fatal cases illustrating how cognitive biases delayed AHTR recognition. Failure of hemoglobin to rise after transfusion should prompt immediate evaluation for hemolysis and reassessment of transfusion compatibility.

**Abbreviations**
AF: atrial fibrillation
AHTR: acute hemolytic transfusion reaction
AKI: acute kidney injury
aPTT: activated partial thromboplastin time
BUN: blood urea nitrogen
DAT: direct antiglobulin test
DIC: disseminated intravascular coagulation
DPL: diagnostic peritoneal lavage
ED: emergency department
HTR: hemolytic transfusion reaction
ICU: intensive care unit
IHN: international haemovigilance network
MTP: massive transfusion protocol
PiCCO: pulse index contour continuous cardiac output
PRBCs: packed red blood cells
PT/INR: prothrombin time/international normalized ratio
RBC: red blood cell
ROWS: rule out worst‐case scenario
SHOT: serious hazards of transfusion
SpO_2_
: peripheral oxygen saturation
WBIT: wrong blood in tube

## 1 Introduction

Trauma management is challenging because of heterogeneous patient profiles, diverse injury patterns, and the urgent need for rapid hemostatic resuscitation [1]. Timely initiation of a massive transfusion protocol (MTP) is critical in patients with exsanguinating hemorrhage [2]. While MTPs are lifesaving, their emergent nature and the large volumes of blood transfused may increase the risk of complications, including hemolytic transfusion reactions (HTRs) [3].

HTRs occur when transfused red blood cells (RBCs) are destroyed prematurely, most often through immune mechanisms involving preformed alloantibodies or anamnestic alloantibody responses [4]. Acute hemolytic transfusion reactions (AHTRs), usually caused by ABO incompatibility, occur during or within 24 h of transfusion and are characterized by intravascular hemolysis [5]. Their severity correlates with the volume of incompatible blood transfused; in such situations, prompt recognition and cessation of transfusion are critical [6]. However, even with rigorous safety protocols, the fast‐paced environment of trauma emergency departments (EDs) managing trauma cases predisposes clinicians to diagnostic and procedural errors [7].

In addition to these clinical and system‐level challenges, cognitive factors may further complicate timely recognition. Cognitive biases, or systematic deviations in reasoning such as anchoring, premature closure, and availability bias, are major contributors to diagnostic errors in emergency medicine and have been implicated in a large proportion of cases of diagnostic error in prior literature [8, 9]. Reports specifically linking these biases to delayed recognition of transfusion reactions in trauma remain limited. Here, we report three cases of ABO‐incompatible AHTRs at a tertiary trauma referral center, in which cognitive biases contributed to delayed recognition of hemolysis and adverse outcomes.

## 2 Case Presentation

### 2.1 Patient 1

A 52‐year‐old man was admitted within 2 h of a motorcycle–car collision. On arrival, he was hypotensive (90/60 mmHg), tachycardic (105 beats/min), and had a Glasgow Coma Scale (GCS) score of 11 (E3V3M5). Imaging demonstrated a right hemopneumothorax, subdural and epidural hematomas, and fractures of the right clavicle and right tibia and fibula. During early resuscitation, he received three units of uncrossmatched packed red blood cells (PRBCs) (approximately 800 mL in total) for traumatic shock attributed mainly to hemothorax and lower‐extremity fractures. However, his hypotension worsened immediately after transfusion. Urgent diagnostic peritoneal lavage was then performed despite normal abdominal and pelvic CT scans. The lavage was negative for intra‐abdominal bleeding. He was admitted to the intensive care unit (ICU) for advanced monitoring and further evaluation of the cause of persistent instability.

Approximately 3 h after initiation of transfusion, and 1 h after completion of the third unit, tea‐colored urine appeared. Hemoglobin fell from 10.5 to 8.0 g/dL, accompanied by rising lactate dehydrogenase (LDH) and indirect bilirubin levels. Coagulation parameters evolved toward disseminated intravascular coagulation (DIC), with a prolonged prothrombin time/international normalized ratio (PT/INR) and activated partial thromboplastin time (aPTT), and an elevated D‐dimer level. At this stage, however, no suspicion of a transfusion reaction was raised. Because signs of traumatic shock persisted, pulse index contour continuous cardiac output (PiCCO) monitoring was performed, demonstrating low systemic vascular resistance, reduced end‐diastolic volume, and a variable cardiac index. These findings suggested mixed shock and prompted consideration of hemolysis.

Further transfusion was withheld immediately, intravenous access was maintained with normal saline, and a transfusion reaction workup was initiated. The direct antiglobulin test (DAT) on a post‐transfusion sample was positive for C3d and weakly positive for IgG. Repeat ABO/RhD typing and crossmatching confirmed the patient's blood group as O RhD‐positive and identified ABO incompatibility with the transfused units. Bedside and blood bank clerical review documented mis‐issuance of AB RhD‐positive RBCs following an emergency request for uncrossmatched blood. Plasma‐free hemoglobin was elevated, and urinalysis showed hemoglobinuria; blood and unit cultures were negative.

He was subsequently supported with compatible O RhD‐positive RBCs as needed, balanced component therapy for coagulopathy, crystalloid resuscitation, and vasopressor support with norepinephrine. Renal‐protective measures were instituted with close urine output monitoring, followed by renal replacement therapy when indicated. Although laboratory markers of hemolysis gradually improved, the patient developed progressive acute kidney injury (AKI) and multiorgan dysfunction and died on hospital day 20. Severe polytrauma remained the principal cause of death; nevertheless, delayed recognition of ABO‐incompatible AHTR, likely contributed to clinical deterioration.

### 2.2 Patient 2

An 81‐year‐old man with end‐stage renal disease, atrial fibrillation (AF), diabetes mellitus, hypertension, and prostate cancer was admitted after a fall resulting in a right femoral neck fracture. He underwent surgical fixation and was transferred to the ICU postoperatively for close monitoring given his advanced age and multiple comorbidities. On ICU admission, the patient developed respiratory distress, which, in combination with his underlying AF, raised suspicion of pulmonary thromboembolism. However, CT pulmonary angiography showed no evidence of pulmonary thromboembolism. The postoperative decline in hemoglobin from 12 g/dL to 8.5 g/dL was initially attributed to surgical blood loss. Four units of crossmatched PRBCs (approximately 1100 mL in total) were transfused within 24 h. Despite repeated transfusions, hemoglobin failed to rise. This prompted evaluation for gastrointestinal bleeding; however, no source of hemorrhage was identified.

Approximately 24–36 h after transfusion, laboratory studies showed elevated LDH and indirect bilirubin levels, progressive oliguria, and worsening azotemia (Blood urea nitrogen [BUN] 51 → 54 mg/dL; creatinine 2.1 → 2.9 mg/dL). Coagulation studies revealed a prolonged PT/INR and aPTT, thrombocytopenia (approximately 35 × 10^9^/L), and a falling fibrinogen level, raising concern for evolving DIC. On ICU day 3, a peripheral blood smear demonstrated prominent schistocytosis (≥ 10%), consistent with microangiopathic hemolysis in the setting of DIC. At that point, an AHTR was strongly suspected. The transfusion was stopped immediately, intravenous access was maintained with normal saline, and a full transfusion reaction workup was initiated.

The DAT was positive (C3d ± IgG). Blood cultures from the patient and cultures of the implicated transfused units were negative. Repeat testing confirmed his true blood group as B RhD‐positive, whereas the admission specimen had been mislabeled as A RhD‐positive, leading to the issuance of incompatible A RhD‐positive units. Urinalysis showed hemoglobinuria, although interpretation was limited by low urine output. The temporal association between incompatible transfusion and subsequent coagulopathy, schistocytosis, and renal injury established severe AHTR with secondary DIC and AKI.

He was subsequently supported with compatible B RhD‐positive or group O RBCs for ongoing needs, balanced component therapy for DIC, crystalloid resuscitation, and vasopressors as indicated. Renal replacement therapy was continued as clinically indicated. Although hemolysis indices partially improved, he developed worsening renal failure, recurrent AF with rapid ventricular response, and multiorgan dysfunction, and died on hospital day 37. While advanced age, several comorbidities, and his postoperative state were major contributors, ABO‐incompatible AHTR likely precipitated the terminal decline.

### 2.3 Patient 3

A 30‐year‐old woman presented after being struck by a car. On arrival, her blood pressure was 105/75 mmHg, heart rate was 90 bpm, respiratory rate was 18 breaths/min, peripheral oxygen saturation (SpO_2_) was 95% on room air, and GCS score was 14/15. CT imaging demonstrated acute subdural and epidural hematomas with a basilar skull fracture. Thoracic injuries included pulmonary contusion and pneumomediastinum. Initial hemoglobin was 9.8 g/dL. During early resuscitation, she received one unit of PRBCs (approximately 250 mL) that had been incorrectly issued as uncrossmatched emergency blood despite not being group O. She subsequently underwent emergent craniotomy, ventriculostomy, and tube thoracostomy, during which two additional units (total incompatible exposure: three units, approximately 800 mL) were transfused under the same emergency request.

Approximately 6 h after transfusion, hemoglobin failed to increase as expected and remained around 10 g/dL. No immediate fever, rigors, hypotension, or bronchospasm were documented. Laboratory evaluation revealed rising LDH, indirect hyperbilirubinemia, and undetectable haptoglobin. Urinalysis was positive for blood on dipstick, with only a few RBCs on microscopy, supporting hemoglobinuria; the RBCs detected microscopically were attributed to concomitant trauma. Coagulation studies were abnormal, with a prolonged PT/INR and aPTT, an elevated D‐dimer, and falling platelet counts, raising concern for incipient DIC.

When hemolysis was suspected, further transfusion was stopped, intravenous access was maintained with normal saline, and a transfusion‐reaction workup was initiated. Bedside and blood bank clerical checks identified a preparation or issuance error associated with the emergency request. Repeat ABO/RhD typing and compatibility testing confirmed the patient's blood group as B RhD‐positive, whereas the transfused units were A RhD‐negative, establishing ABO‐incompatible exposure. The DAT on a post‐transfusion sample was positive (C3d ± IgG). Blood cultures from the patient and cultures of the implicated units were negative and did not support a septic transfusion reaction.

She was supported with B RhD‐positive or group O RhD‐compatible RBCs, balanced component therapy for coagulopathy, crystalloids, vasopressors as required, and intensive neurocritical care measures. Despite partial improvement in hemolysis indices, her course was complicated by progressive neurologic decline, secondary sepsis, and multiorgan dysfunction and she died on hospital day 16. The combined effects of severe head trauma and delayed recognition of AHTR were considered contributory to the fatal outcome.

## 3 Discussion

Hemorrhage is the leading cause of preventable death in trauma patients. Each minute of delay in activating MTP increases mortality by approximately 5%, independent of product ratios [2, 10, 11, 12]. Guidelines recommend prompt damage‐control resuscitation and MTP activation when patients present with abnormal vital signs such as an elevated shock index or low systolic blood pressure [12, 13, 14]. The emphasis on early transfusion has extended to prehospital settings [15, 16, 17]. Although the benefits of RBC transfusions generally outweigh the risks, AHTRs can still occur in trauma centers [3, 18, 19].

Hemolysis in AHTRs is typically intravascular and is most often caused by ABO incompatibility [5]. Complement‐mediated RBC lysis releases free hemoglobin into the circulation and may precipitate shock, AKI, and DIC [20]. Clinical manifestations include back or flank pain, fever, chills, hypotension, hemoglobinuria, oliguria or anuria, and bleeding related to DIC, such as epistaxis or oozing at intravenous access sites [21]. The incidence of HTRs and associated fatalities varies across settings [22, 23, 24]. The 2024 Serious Hazards of Transfusion (SHOT) report from the United Kingdom documented 51 HTRs following more than 1.6 million RBC transfusions, including 16 AHTRs and three deaths [24]. International Haemovigilance Network (IHN) data from 2006‐2012 showed that AHTRs accounted for 6.9% of 349 transfusion‐associated deaths [22]. In trauma settings, uncrossmatched transfusions and procedural errors such as wrong blood in tube (WBIT) can precipitate an AHTR [18, 19]. One review found that among emergency recipients of uncrossmatched RBCs, 3.7% had preexisting alloantibodies, 0.6% received incompatible units, and 0.06% developed an AHTR [18]. Although precise center‐specific incidence data were not systematically collected, ABO‐incompatible AHTRs appears to be exceedingly rare at our institution relative to the large number of trauma transfusions performed annually.

Comparison with previous trauma‐related reports helps contextualize the contribution of the present study. Fiorellino et al. reported a single trauma patient who developed acute hemolysis, DIC, and renal failure after incompatible uncrossmatched transfusion during resuscitation [18]. Davis et al. described a similar single‐case scenario in which a blood‐typing error during massive transfusion led to a hemolytic reaction and highlighted preventive workflow measures [3]. More recently, Krishna et al. reported a polytrauma patient with WBIT‐related incompatible transfusion in whom hemolysis was initially difficult to distinguish from trauma‐related complications [19]. In contrast, our case series identifies a consistent pattern across three fatal cases and explicitly analyzes cognitive biases as contributors to delayed recognition, thereby extending the literature beyond procedural error alone.

Rapid recognition is crucial because severity increases with the volume of incompatible blood transfused [6]. An unrecognized AHTR may trigger DIC, cause uncontrolled bleeding, worsen shock, and lead to further incompatible transfusions [4]. Distinguishing hemolysis from trauma‐related anemia, coagulopathy, or sepsis, however, is difficult [18, 19]. Across the three cases, persistent anemia or failure of hemoglobin to rise and evolving coagulopathy were initially attributed to injury‐related or perioperative causes. Other findings, including hemoglobinuria and rising LDH and indirect bilirubin levels, did not immediately prompt evaluation for hemolysis; this diagnostic framing delayed recognition. Beyond clinical and procedural factors, cognitive mechanisms also play a critical role in delayed recognition. Dual‐process theory, widely used to explain diagnostic reasoning, distinguishes two modes of reasoning: System 1 is fast and intuitive and generates hypotheses through pattern recognition. System 2 is slower and more analytical and relies on deliberate reasoning [25]. In high‐pressure critical care environments such as ICUs and EDs, clinicians must make rapid decisions under uncertainty [26, 27]. Heuristics, or mental shortcuts, within System 1 conserve time and cognitive resources but may also be vulnerable to cognitive biases [28, 29, 30]. These biases represent systematic deviations from optimal reasoning and may lead to diagnostic error [29]. Prior studies have estimated that diagnostic errors affect 0.6% to 12% of first‐time ED patients, with cognitive factors implicated in the majority of such cases [9]. Availability, confirmation, anchoring, and premature closure are among the most commonly described cognitive biases in EDs [9, 31, 32, 33]. Additional errors such as search satisficing and diagnostic momentum have also been described in emergency care [28, 34]. Table 1 maps these biases to the manifestations observed in our cases and underscores how urgency and handoffs may amplify them beyond the ED.

**TABLE 1:** Cognitive biases relevant to delayed recognition of AHTRs in our cases.

Bias	Definition	Manifestations in our cases
Availability bias	Judging a diagnosis as more likely because it is recent, familiar, or vivid in memory [35]	Recent exposure to trauma‐related hemorrhagic shock biased clinicians toward bleeding as the cause of instability
Anchoring bias	Overreliance on an initial impression despite subsequent conflicting data [36]	Teams remained fixed on hemorrhagic shock despite non‐rising hemoglobin and post‐transfusion hemoglobinuria
Search satisficing	Stopping further diagnostic inquiry after identifying an initial plausible explanation [28]	Once trauma‐related bleeding was assumed, evaluation for hemolysis was delayed
Premature closure	Accepting a diagnosis before adequate verification and excluding key alternatives [37]	Hemorrhagic shock was accepted without adequately excluding transfusion reaction
Diagnosis momentum	Persistence of an initial diagnostic label across handoffs and team transitions [34]	The label of “trauma‐related shock” persisted into ICU care, lowering suspicion for AHTR
Confirmation bias	Focusing on findings that support the leading diagnosis while discounting conflicting evidence [38]	Investigations were directed toward confirming bleeding rather than reconsidering hemolysis

Abbreviations: AHTR, acute hemolytic transfusion reaction; ICU, intensive care unit.

This study illustrates that, although early MTP activation is essential, the urgency of trauma care may amplify cognitive errors, some of which persist beyond the ED and delay recognition of AHTRs. Our experience also highlights the potential value of what we term an “ICU secondary survey,” defined as a structured reassessment performed after initial stabilization that explicitly revisits discordant clinical findings (e.g., failure of hemoglobin to rise after transfusion, new hemoglobinuria, or evolving coagulopathy). In our cases, this step was effectively omitted, which may have contributed to delayed recognition of the reactions. To improve consistency, this process can be operationalized as a checklist‐based approach that includes: [1] verification of the transfusion history and compatibility, with cross‐checking of patient identity and blood group; [2] targeted evaluation for hemolysis using repeat hemoglobin measurement, LDH, indirect bilirubin, haptoglobin, and urinalysis; [3] systematic reassessment of alternative causes of instability (e.g., occult bleeding, sepsis, or other shock states); and [4] emphasis on laboratory trends rather than isolated values, particularly nonresponsehemoglobin to transfusion. In parallel, a brief diagnostic time‐out triggered by predefined red flags can prompt clinicians to reconsider initial assumptions, generate alternative diagnoses, and verify critical data. Checklist‐based reassessment and cognitive forcing strategies have been shown to reduce diagnostic error and improve clinical reasoning in high‐risk settings [39, 40, 41].

Beyond bedside considerations, ABO‐incompatible transfusions often arise from systems‐level failures rather than isolated clinical errors. Hemovigilance data consistently identify WBIT events, failures of patient identification, and errors in blood‐component selection or administration as leading causes [23, 42]. Human factors, particularly slips and lapses under high workload, interruptions, and protocol deviations, substantially contribute to these events [42]. Additional vulnerability arises from inadequate redundancy, including failures of second‐sample or independent verification [23]. Limitations in electronic systems, such as suboptimal integration of transfusion workflows and inconsistent use of barcode‐based identification, may further increase risk [43, 44]. Some of these mechanisms align with the errors observed in our cases and highlight the need for standardized verification processes and robust system‐level safeguards in high‐acuity settings.

**FIGURE 1:**
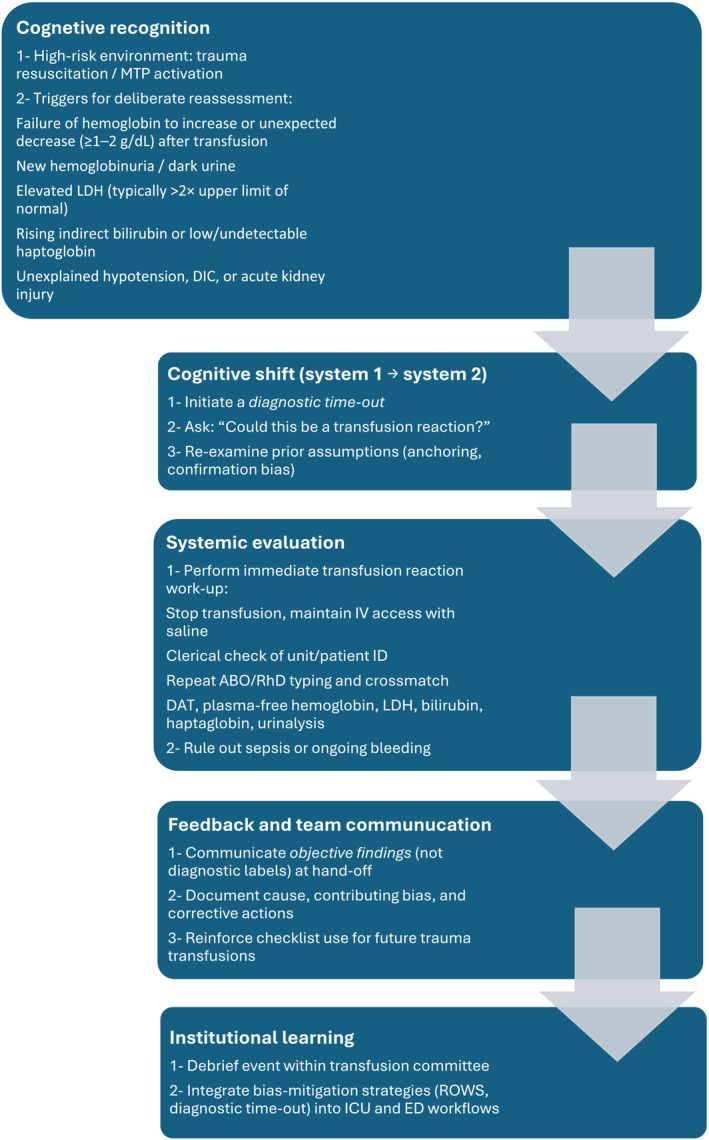
Stepwise framework for recognition and management of acute hemolytic transfusion reactions (AHTRs) during trauma resuscitation. The figure outlines key triggers for reassessment, structured diagnostic steps, and system‐level responses to reduce delays in recognition. Abbreviations: DAT, direct antiglobulin test; DIC, disseminated intravascular coagulation; ED, emergency department; ICU, intensive care unit; ID, identification; IV, intravenous; LDH, lactate dehydrogenase; MTP, massive transfusion protocol; ROWS, rule out worst‐case scenario.

Multiple strategies may mitigate cognitive errors, most of which are grounded in dual‐process theory. The central aim is to trigger a shift from System 1 to System 2 reasoning when the risk of error is high [40, 45]. Building a foundational understanding of decision‐making theory and error mechanisms can help clinicians recognize when such a shift is needed. Targeted education on specific biases may foster “bias inoculation” and prepare physicians to anticipate predictable pitfalls, as many clinical scenarios involve cognitive biases that contribute to diagnostic error [34, 39, 40, 46]. Identifying predefined red flags, for example, failure of hemoglobin to rise despite transfusion or unexpected hemoglobinuria, may be particularly useful in trauma resuscitation and early ICU care. Our suggested steps for reducing cognitive error in the evaluation of suspected AHTR following emergency trauma transfusion are outlined in Figure 1.

Cognitive biases often interact dynamically: for example, anchoring and search satisficing may lead to premature closure, while confirmation bias may emerge within the context of premature closure [46]. Interventions aimed at one bias may therefore reduce error at multiple points. Table 2 summarizes practical mitigation techniques tailored to the patterns observed in our patients. Helping clinicians identify their own biases through structured reflection may further promote corrective strategies.

**TABLE 2:** Cognitive‐bias mitigation strategies relevant to AHTR recognition in trauma.

Cognitive bias	Mitigation strategy	Application to our cases
Availability bias	Prioritize case‐specific evidence over recent or familiar clinical patterns [34]	Hemoglobinuria and poor hemoglobin response should have prompted reconsideration of hemolysis rather than recurrent hemorrhage
Anchoring bias	Use a brief diagnostic time‐out to reassess assumptions and verify data consistency [34, 46]	A pause after transfusion non‐response could have redirected attention from hemorrhagic shock to possible AHTR
Search satisficing	Use structured checklists to ensure alternative diagnoses are actively reconsidered [28, 46]	A checklist including hemolysis markers could have triggered an earlier AHTR workup
Premature closure	Adopt a “not yet diagnosed” or ROWS approach until critical alternatives are excluded [34, 46]	New hemoglobinuria, DIC, or renal dysfunction should have prompted reopening the differential diagnosis
Diagnostic momentum	Communicate objective findings rather than inherited diagnostic labels during handoffs [34]	Reporting persistent anemia, coagulopathy, and hemoglobinuria may have prompted reconsideration of AHTR in the ICU
Confirmation bias	Seek disconfirming evidence and invite alternative interpretations or second opinions [34]	Negative imaging findings or lack of ongoing bleeding should have shifted attention toward transfusion‐related hemolysis

Abbreviations: AHTR, acute hemolytic transfusion reaction; DIC, disseminated intravascular coagulation; ICU, intensive care unit; ROWS, rule out worst‐case scenario.

Finally, many recommended techniques, including obtaining a thorough history, performing a careful examination, and maintaining a broad differential diagnosis, are core clinical skills. Yet the chaotic ED environment, heavy workload, operational stressors, and fatigue that contribute to cognitive biases can also hinder the shift to system 2 thinking [28, 46]. Our case series underscores the rarity of ABO‐incompatible AHTR, the risk of delayed or missed recognition in trauma pathways, and the potential value of deliberate, structured reassessment in the ICU. Prospective studies should evaluate whether debiasing tools and a structured ICU secondary survey can improve the timeliness of AHTR recognition and patient outcomes without compromising the benefits of early transfusion.

## 4 Conclusion

In trauma centers, AHTRs may remain undetected because their manifestations overlap with those of trauma‐related complications. Cognitive biases such as anchoring, search satisficing, premature closure, confirmation bias, and diagnostic momentum may further delay recognition and worsen outcomes. Integrating cognitive awareness with structured clinical approaches, including diagnostic time‐outs, targeted reassessment, and standardized checklists, could facilitate earlier detection. Addressing cognitive factors in trauma transfusion practice should therefore be considered alongside optimization of transfusion protocols and warrants further prospective evaluation.

## Author Contributions

**Mohammad Javad Entezari Meybodi:** conceptualization, validation, writing – original draft. **Samin Jodari Dallali:** investigation, writing – review and editing. **Hosein Kouchaki:** data curation, supervision, visualization, writing – original draft. **Golnar Sabetian:** project administration, writing – review and editing.

## Funding

The authors did not receive any specific funding for this study.

## Ethics Statement

Written informed consent for participation, as approved by the Ethics Committee of Shiraz University of Medical Sciences, was obtained from the first‐degree family members of the patients because they had died.

## Consent

As the patients described in this case series were deceased, written informed consent for the publication of their anonymized clinical data was obtained from their next of kin. All patient information was deidentified to ensure privacy and compliance with ethical standards.

## Conflicts of Interest

The authors declare no conflicts of interest.

## Data Availability

All the data generated or analyzed during this study are included in this published article.
